# Bone vitality and vascularization of mandibular and maxillary bone grafts in maxillary sinus floor elevation: A retrospective cohort study

**DOI:** 10.1111/cid.13142

**Published:** 2022-10-10

**Authors:** Vivian Wu, Engelbert A. J. M. Schulten, Marco N. Helder, Christiaan M. ten Bruggenkate, Nathalie Bravenboer, Jenneke Klein‐Nulend

**Affiliations:** ^1^ Department of Oral Cell Biology, Academic Centre for Dentistry Amsterdam (ACTA) University of Amsterdam and Vrije Universiteit Amsterdam, Amsterdam Movement Sciences Amsterdam The Netherlands; ^2^ Department of Oral and Maxillofacial Surgery/Oral Pathology, Amsterdam UMC and Academic Centre for Dentistry Amsterdam (ACTA) Vrije Universiteit Amsterdam, Amsterdam Movement Sciences Amsterdam The Netherlands; ^3^ Department of Clinical Chemistry, Amsterdam UMC Vrije Universiteit Amsterdam, Amsterdam Movement Sciences Amsterdam The Netherlands

**Keywords:** autografts, bone regeneration, bone vitality, mandibular bone, maxillary bone, maxillary sinus floor elevation, vascularization

## Abstract

**Objectives:**

Mandibular retromolar (predominantly cortical) and maxillary tuberosity (predominantly cancellous) bone grafts are used in patients undergoing maxillary sinus floor elevation (MSFE) for dental implant placement. The aim of this retrospective cohort study was to investigate whether differences exist in bone formation and vascularization after grafting with either bone source in patients undergoing MSFE.

**Methods:**

Fifteen patients undergoing MSFE were treated with retromolar (*n* = 9) or tuberosity (*n* = 6) bone grafts. Biopsies were taken 4 months postoperatively prior to dental implant placement, and histomorphometrically analyzed to quantify bone and osteoid area, number of total, apoptotic, and receptor activator of nuclear factor‐κB ligand (RANKL)‐positive osteocytes, small and large‐sized blood vessels, and osteoclasts. The grafted area was divided in three regions (caudal‐cranial): RI, RII, and RIII.

**Results:**

Bone volume was 40% (RII, RIII) higher and osteoid volume 10% (RII) lower in retromolar compared to tuberosity‐grafted areas. Total osteocyte number and number of RANKL‐positive osteocytes were 23% (RII) and 90% (RI, RII) lower, but osteoclast number was higher (retromolar: 12, tuberosity: 0) in retromolar‐grafted areas. The total number of blood vessels was 80% (RI) to 60% (RIII) lower, while the percentage of large‐sized blood vessels was 86% (RI) to 25% (RIII) higher in retromolar‐grafted areas. Number of osteocyte lacunae and apoptotic osteocytes were similar in both bone grafts used.

**Conclusions:**

Compared to the retromolar bone, tuberosity bone showed increased bone vitality and vascularization in patients undergoing MSFE, likely due to faster bone remodeling or earlier start of new bone formation. Therefore, tuberosity bone grafts might perform better in enhancing bone regeneration.

**What is known:**

- Cortical bone grafts are considered to have less bone regeneration potential than cancellous bone grafts, due to lack of osteogenic cells and less osteoconductive matrix surface.
- Retromolar (cortical) and tuberosity (cancellous) bone grafts are used in patients undergoing maxillary sinus floor elevation (MSFE) for dental implant placement, but their bone regeneration potential has not been compared.

**What this study adds:**

- Tuberosity bone grafts show enhanced bone vitality and vascularization in MSFE compared to retromolar bone grafts.
- Tuberosity bone grafts result in more osteoid deposition, blood vessel formation, and active bone remodeling, indicating that tuberosity bone might perform better as autologous graft in MSFE than retromolar bone.

## 1 INTRODUCTION

Maxillary sinus floor elevation (MSFE) is a frequently performed surgical procedure to restore insufficient bone height in the posterior maxilla allowing dental implant placement.^1^, ^2^, ^3^, ^4^ In MFSE, the space created between the maxillary alveolar process, the elevated Schneiderian membrane, and the inwardly rotated lateral sinus wall is filled with graft material. Autologous bone is considered as the gold standard grafting material in MSFE,^5^, ^6^ due to its osteoconductive as well as osteoinductive properties. Moreover, it contains osteogenic cells, and does not evoke immunogenic responses. Histologically, autologous bone grafts in MSFE result in predominantly a mature, lamellar type regenerated bone with higher mineralized bone volumes compared to bone substitutes which result in regenerated bone with lower mineralized bone volumes with a more immature, woven type of bone.^7^, ^8^, ^9^ Therefore, autologous bone demonstrates increased bone regenerative potential compared to other grafting materials, such as synthetic, xenograft, or allograft bone substitutes with only osteoconductive properties.^5^

Various donor sites are available to harvest autologous bone, including iliac crest, calvaria, tibia, and intraoral sites (mandible, maxilla).^10^, ^11^, ^12^, ^13^ The choice of the donor site is based on the type and quantity of bone graft required, the ease of access to the donor site, and the time required with regard to the harvesting procedure and costs involved.^3^, ^12^, ^13^, ^14^, ^15^ Autologous bone grafts from intraoral sources are widely used in MSFE, either applied purely or mixed with a bone substitute.^3^ A major advantage of intraoral sites for bone harvesting compared to extraoral sites, is that the graft can be harvested under local anesthesia.^13^, ^14^ The mandibular retromolar and maxillary tuberosity regions are favorable donor sites due to low morbidity compared to other intraoral sites.^13^, ^14^, ^16^ There are multiple clinical and biological differences between bone harvested from the retromolar versus the tuberosity region. Bone from the retromolar region is predominantly cortical with a high mineral density, while bone from the tuberosity is more cancellous with a lower mineral density.^16^, ^17^

Cortical bone grafts are considered to have less bone regeneration potential than cancellous bone grafts, due to the lack of osteogenic bone marrow cells and less osteoconductive matrix surface.^18^, ^19^, ^20^ Cortical bone grafts show delayed vascularization due to their lack of porosity and consequent inhibition of vascular ingrowth, resulting in reduced diffusion of oxygen and nutrients through the cortical matrix. Therefore, cells in cortical grafts, compared to cancellous grafts, are less likely to survive grafting procedures. It has been suggested that primitive osteogenic cells surviving transplantation and forming mature osteoblasts are crucial for the formation of new bone.^20^, ^21^, ^22^ Moreover, cortical bone grafts contain fewer osteoprogenitors than cancellous bone. Finally, the remodeling period of cortical bone graft takes longer, due to longer resorption time preceding osteogenic new bone formation.^21^, ^22^

The majority of histologic and histomorphometric studies evaluating different sites and methods of autologous bone grafting in MSFE investigated bone grafts from the iliac crest and chin.^12^ Only four studies investigated purely retromolar bone graft in MSFE.^8^, ^23^, ^24^, ^25^ No studies investigated purely tuberosity bone graft in MSFE. Comparison of the bone regeneration potential of retromolar bone grafts with tuberosity bone grafts in MSFE by means of histological and histomorphometrical analysis has not been performed so far.

Therefore, this study aimed to investigate possible differences in bone vitality and vascularization in patients undergoing MSFE using retromolar or tuberosity bone grafts through histomorphometrical analysis of bone biopsies. Four months after the MSFE, we evaluated the biopsies prior to dental implant placement. It was hypothesized that tuberosity compared to retromolar bone graft will show enhanced new bone formation in patients undergoing MSFE. In this study, we report the first comparison of retromolar and tuberosity bone grafts for bone vitality and vascularization in patients undergoing MSFE.

## 2 MATERIALS AND METHODS

### 2.1 Study approval

The protocol was approved by the medical ethics committee (IRB) of the VU University Medical Center in Amsterdam (#2016.105). All patients signed a written informed consent before participation in the study. The study was performed according to the STROBE guidelines.^26^

### 2.2 Patient selection

Fifteen patients (4 females and 11 males), who were partially edentulous in the posterior maxilla and required dental implants for prosthetic rehabilitation between 2003 and 2012 were selected consecutively for this study (Table 1). All patients required an MSFE due to insufficient vertical bone height (≤3 mm) in at least one of the planned dental implant positions. Since some biopsies of these locations broke apart and could not be reconstructed properly, we sometimes had to switch to adjacent biopsies instead. The average age of the patients was 56 ± 2 years (mean ± SEM). Nine patients undergoing MSFE received mandibular retromolar bone graft, and six patients received a maxillary tuberosity bone graft. The average residual bone height was 6 ± 1 mm (mean ± SEM), with an average residual bone height in patients grafted with retromolar bone of 5 ± 1 mm (mean ± SEM) and with tuberosity bone of 7 ± 1 mm (mean ± SEM). Patient demographics are summarized in Table 1.

**TABLE 1:** Patient data

Donor site	Gender (♂,♀)	Age (years)	Residual bone height (mm)	Dental implant position	Biopsy location
Retromolar	♀	44	3	16	Single gap
Retromolar	♂	49	1	27	Multiple gap
Retromolar	♂	54	11	24	Free‐ending
Retromolar	♂	55	4	16	Free‐ending
Retromolar	♂	62	7	25	Free‐ending
Retromolar	♂	67	1	16	Free‐ending
Retromolar	♂	67	6	14	Free‐ending
Retromolar	♂	60	6	16	Free‐ending
Retromolar	♂	53	5	26	Free‐ending
Tuberosity	♂	67	1	25	Multiple gap
Tuberosity	♀	50	10	24^a^	Free‐ending
Tuberosity	♀	50	9	25^a^	Free‐ending
Tuberosity	♀	35	6	17	Free‐ending
Tuberosity	♂	65	8	25	Free‐ending
Tuberosity	♀	58	9	26	Free‐ending
Tuberosity	♂	61	7	15^b^	Free‐ending
Tuberosity	♂	61	5	16^b^	Free‐ending

*Note*: Gender, age, residual bone height, dental implant position, biopsy location in patients undergoing MSFE treated with mandibular retromolar or maxillary tuberosity bone grafts.

^a^ Same patient.

^b^ Same patient.

The patients included in this study had a healthy periodontium and were non‐smokers or moderate smokers (<10 cigarettes/day). Patients who required horizontal bone augmentation, and patients with specific conditions, for example, systemic diseases, drug abuse, heavy smokers, other semi‐invasive dental treatments, and/or pregnancy, were not included in this study. One oral and maxillofacial surgeon performed all surgical procedures both in the Alrijne Hospital, Leiderdorp, and in Amsterdam UMC, location VUmc, Amsterdam, The Netherlands.

### 2.3 Maxillary sinus floor elevation

**FIGURE 1:**
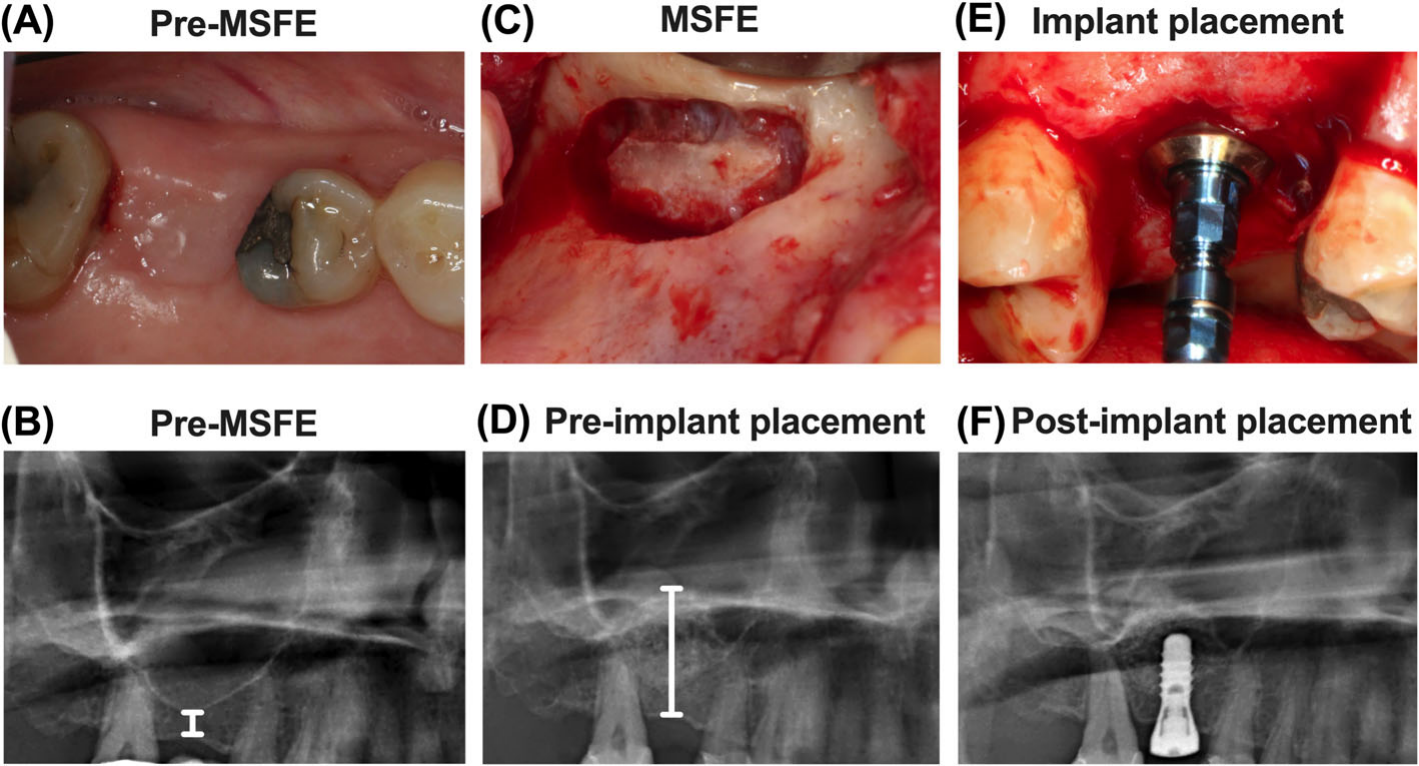
Clinical photographs of maxillary sinus floor elevation (MSFE) via a lateral approach allowing dental implant placement, and their corresponding radiographs to evaluate maxillary sinus and alveolar bone height. (A) Preoperative photograph of the clinical situation. (B) Preoperative radiograph of the maxillary sinus. White line: residual native bone height. (C) Photograph of the lateral window during MSFE. (D) Radiograph of the maxillary sinus 4 months post‐MSFE prior to implant placement. White line: total bone height. (E) Photograph of implant placement at 4 months post‐MSFE. (F) Radiograph of the maxillary sinus directly after implant placement.

All 15 patients underwent MSFE as previously described.^2^ A preoperative clinical photograph (Figure 1A) and a radiograph (Figure 1B) were taken, and a lateral bony window was prepared and turned inward and upward leaving the lifted Schneiderian membrane intact (Figure 1C). The generated cavity within the maxillary sinus was filled with pure autologous bone harvested from either the retromolar or tuberosity region. Wound closure was performed with Gore‐Tex sutures (W.L. Gore and Associates, Newark, DE, USA), which were removed after 10–14 days. All patients received antibiotic prophylaxis, consisting of 500 mg amoxicillin, 3 times daily starting 1 day preoperatively and continuing 7 days postoperatively. After a healing period of 4 months (post‐MSFE), prior to dental implant placement, a panoramic radiograph was made to determine the increase in vertical tissue height at the planned dental implant positions (Figure 1D).

### 2.4 Autologous bone graft harvesting technique

The retromolar bone grafts were harvested in half‐cylinder shape with explantation trephines (inner diameter 4.2 mm; Institute Straumann AG, Basel, Switzerland), with a drilling speed of 500 rpm with minimal pressure and using sterile saline for copious irrigation, from the external oblique ridge of the mandible. The harvested half‐cylinder bone cores were used as a cylinder to fill the recipient site. The half‐cylinders were not milled but placed as such in the maxillary sinus bottom. The maxillary tuberosity bone grafts were harvested with hammer and osteotome. The harvested bone pieces were cut with a bone rongeur into smaller pieces to fill the recipient site. Wound closure was performed with resorbable sutures.

### 2.5 Dental implant surgery

Four months after MSFE, dental implant surgery was performed under local anesthesia (Figure 1E). A crestal incision was made with mesial and distal buccal vertical release incisions. A full‐thickness mucoperiosteal flap was raised to expose the underlying alveolar ridge, which was inspected visually for sufficient bone volume for the intended dental implant placement. Bone biopsies were obtained during dental implant surgery, using trephine drills with a length of 40.5 mm, and with an external diameter of 3.5 mm matching the outer core diameter of the dental implants and an inner diameter of 2.5 mm (Institute Straumann AG, Basel, Switzerland), with a drilling speed of 500 rpm and using sterile saline for copious irrigation, prior to dental implant insertion. Immediately after dental implant placement, a panoramic radiograph was made to check dental implant positions (Figure 1F). Panoramic radiographs taken pre‐MSFE, as well as before dental implant placement, were used for morphometric measurements to determine the increase in vertical tissue height at the planned dental implant positions, using digital software. Calculations were performed with the use of a conversion factor (1.25×) that adjusted for magnification of the panoramic radiograph. After 3 months of osseointegration of the dental implants, the superstructures were fabricated and placed by the patient's dentist.

### 2.6 Bone biopsies

**FIGURE 2:**
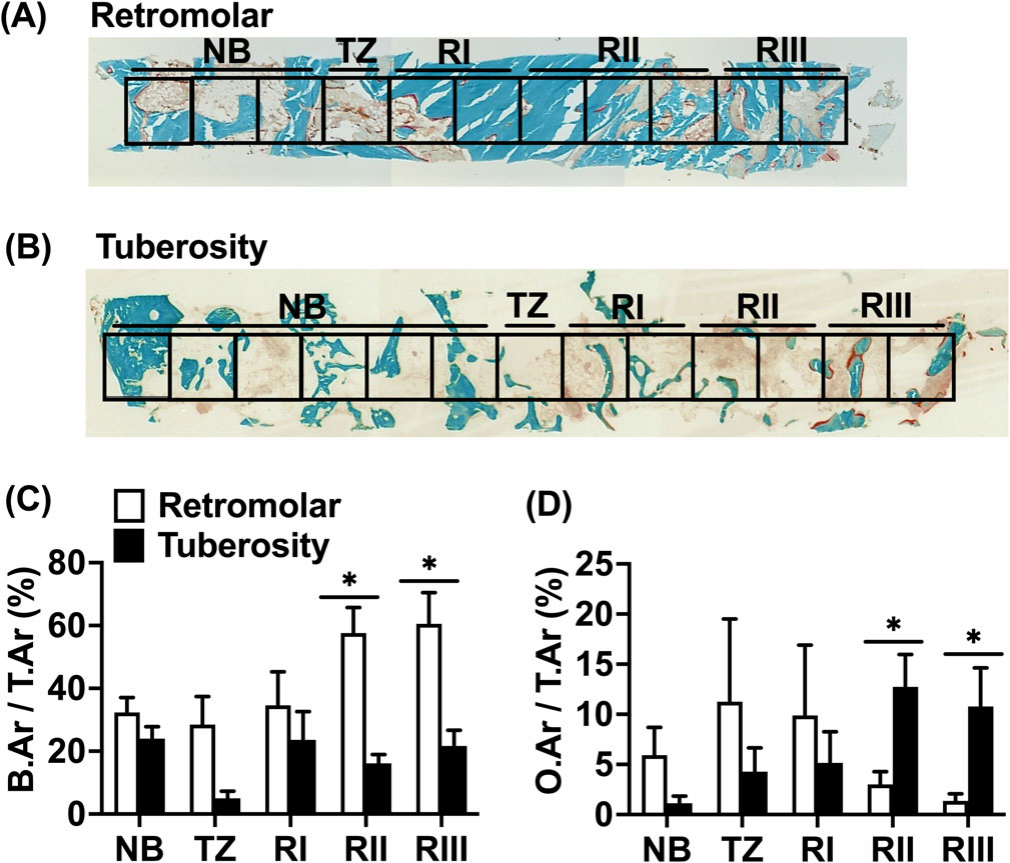
Histomorphometric analysis of biopsies taken after maxillary sinus floor elevation (MSFE) with retromolar or tuberosity bone graft. (A) Representative biopsy from one patient after MSFE with retromolar bone graft. (B) Representative biopsy from one patient after MSFE with tuberosity bone graft. Midsagittal histological sections of each biopsy were stained with Goldner's trichome method, to distinct mineralized bone tissue (green) and unmineralized osteoid (red). Biopsies were divided in consecutive 1 mm^2^ regions of interest (ROIs). The transition zone (TZ) indicated the first ROI where graft material was observed. The images illustrated more bone and less osteoid in retromolar than in tuberosity bone biopsies (RII and RIII regions). Original magnification: 100×. (C) Bone volume (bone area over total area [B.Ar/T.Ar%]), and (D) Osteoid volume (osteoid area over total area [O.Ar/T.Ar%]) as assessed by histomorphometrical analysis. B.Ar/T.Ar and O.Ar/T.Ar were assessed for NB, TZ, Rl, Rll, and Rlll. Values are mean ± SEM (*n* = 3–9). *Significantly different between retromolar and tuberosity bone graft, *p* < 0.05. B.Ar, bone area; NB, native bone; O.Ar, osteoid area; RI, region I; RII, region II; RIII, region III; T.Ar, total area; TZ, transition zone.

The bone biopsies taken during dental implant surgery with a trephine drill were fixated in 4% phosphate‐buffered formaldehyde solution (Klinipath BV, Duiven, The Netherlands) for at least 24 h. The bone biopsies were carefully removed from the trephine burr by cutting the burr, and opening it. Thereafter the bone biopsies were transferred to 70% ethanol, and stored until use for histomorphometrical analysis, as described below under “Histology and histomorphometry.” The caudal side of the bone biopsy had a sharp cutting edge in contrast to a dome‐shaped, crumbled cranial side (Figure 2A,B). These histologic features were used to identify the apicocoronal orientation of the biopsy. Subsequently, the whole research team verified whether the apicocoronal orientation of the biopsy corresponded with the histological appearance. Consensus was reached for all specimens.

Seventeen biopsies from gap, multiple gap, and free‐ending locations were evaluated (Table 1). The following biopsy location definitions were used: (1) single gap location: a natural tooth is present at both sides of the dental implant location; (2) multiple gap location: a natural tooth is present on either side of at least two dental implants next to each other; multiple bone biopsies can be retrieved in this type of gap; and (3) free‐ending location: there is only one natural tooth present at the mesial side of the dental implant location(s); multiple bone biopsies can be retrieved in this situation.

### 2.7 Histology and histomorphometry

After dehydration in descending alcohol series, the bone specimens were embedded without prior decalcification in low‐temperature polymerizing methylmethacrylate (MMA, Merck Schuchardt OHG, Hohenbrunn, Germany). Longitudinal sections of 5 μm thickness were prepared using a Jung K (R. Jung, Heidelberg, Germany) or Polycut 2500 S microtome (Leica, Wetzlar, Germany). Midsagittal histological sections of each biopsy were stained with Goldner's Trichome method,^27^ to distinguish mineralized bone tissue (green) and unmineralized osteoid (red). The histological sections were divided into multiple regions of interest (ROI) of 1 mm^2^ for blinded histomorphometrical analysis, as previously described.^28^ Depending on the length of the biopsy, the number of ROIs ranged from 5 to 15. Vertical tissue height measurements of the residual native bone and graft at the planned dental implant position on the panoramic radiograph were made pre‐MSFE, as well as prior to dental implant placement (Figure 1D). The vertical tissue height of the residual native bone on the radiographs resembled the height of the residual native bone in the biopsy. The vertical tissue height of the graft on the radiographs resembled the height of the graft in the biopsy. The whole research team verified whether the radiographically determined transition zone corresponded with the histological appearance, including parameters such as the occurrence of apoptotic osteocytes and empty osteocyte lacunae to identify grafted material. The consensus was reached for all specimens. The transition zone (TZ) indicates the first ROI where graft material was observed when analyzing from the caudal to the cranial side of the biopsy. Since the biopsies analyzed had different lengths, we decided to define them in three regions after the transition zone (TZ). The first two ROIs on the right of the transition zone were defined as Region I (RI), the two or three ROIs in the center (even or odd numbers) as Region II (RII), and the two most cranial ROIs as Region III (RIII). The digital images of the scanned biopsies were analyzed, starting from the caudal side of the biopsy, and continuing towards the cranial side. This previously described method allowed us to compare similar regions for all biopsies with respect to the bone regeneration and blood vessel formation in the augmented maxillary sinus.^28^, ^29^, ^30^

For each separate area of interest, the histomorphometrical measurements were performed with a computer using an electronic stage table and a Leica DC 200 digital camera (Leica, Wetzlar, Germany). The computer software used was Leica QWin© (Leica Microsystems Image Solutions, Rijswijk, The Netherlands) or NIS‐Elements AR 4.10.01 (Nikon GmbH, Düsseldorf, Germany) at 40× magnification according to the ASBMR nomenclature^31^ to acquire digital images. Bone volume (bone area over total tissue area; B.Ar/T.Ar%) and osteoid volume (osteoid area over bone area; O.Ar/B.Ar%) were calculated as previously described.^32^ The total number of lacunae over bone area (N.Tt.Lac/B.Ar n mm^−2^) and the total number of osteocytes over total number of lacunae (N.Ot/N.Tt.Lac%) were calculated. Only sharp and clearly displayed lacunae with and without osteocytes were included for analysis.

Blood vessel numbers, taking into account the blood vessel size, were determined as mean value of two separate blinded counts. Blood vessel size was calculated as the total blood vessel area expressed in μm^2^. According to their diameter, blood vessels were divided into small (0–400 μm^2^) or large vessels (>400 μm^2^).

Tartrate‐resistant acid phosphatase (TRAcP) staining was used to visualize bone resorbing multinuclear cells (osteoclasts) and was performed on a subset of biopsies (*n* = 6) These sections were selected adjacent to biopsy sections that were stained with Goldner's trichrome method. TRAcP staining was performed according to a standardized protocol.^33^ Quantitative analysis of the number of TRAcP‐positive osteoclasts was carried out at 200× magnification throughout the biopsies according to the previously described ROIs, overlapping with the optical areas in the Goldner's trichrome‐stained sections as closely as possible, using the same computer software and microscope used for quantification of the other histomorphometric parameters in the Goldner trichrome‐stained sections. Within each area, the total number of TRAcP‐positive osteoclasts over bone area (N.Ocl/B.Ar) was calculated.

### 2.8 Immunohistochemistry

A previously described protocol for immunostaining was used.^28^, ^29^, ^34^ To visualize and calculate the number of apoptotic osteocytes, immunohistochemical staining for Cleaved Caspase‐3 was carried out on a subset of biopsies (*n* = 6). Receptor activator of nuclear factor‐κB ligand (RANKL) expression by osteocytes was also detected by immunohistochemistry on a subset of biopsies (*n* = 6). Bone sections embedded in MMA (see “histology and histomorphometry”) were treated with xylene/chloroform (Merck, Darmstadt, Germany) to remove MMA. Sections were rehydrated and endogenous peroxidase quenched with 3% H_2_O_2_ in phosphate‐buffered saline (PBS) containing 40% methanol. Antigen retrieval was performed by incubation with 0.5% saponin (Sigma, St. Louis, MO) in PBS for 30 min, followed by incubation with 3.5 μg/ml DNAse II (Sigma) in a mixture of 25 mM Tris with 10 mM MgSO_4_ for 10 min at room temperature. Then sections were incubated with 3% H_2_O_2_ in PBS containing 40% methanol to block endogenous peroxidase. Nonspecific binding of immunoglobulin G was blocked by incubation with 5% normal goat serum in PBS containing 0.05% Tween. Incubation with primary antibody was performed overnight at 4°C with 1/1000 rabbit‐antiCleaved Caspase‐3 antibody (Cell Signaling Technology, Beverly, MA), or rabbit‐antiRANKL antibody (Cell Signaling Technology, Beverly, MA) in PBS containing 0.05% Tween. Sections were then incubated for 1 h with 1/200 biotin‐labeled goat‐anti‐rabbit immunoglobulin G (Vector Labs, Burlingame, CA) in PBS containing 0.05% Tween, and for 10 min with a Biotin XX Tyramide SuperBoost™ Kit (Thermo Fisher Scientific). For color development, sections were incubated with DAB‐nickel substrate. Sections with Cleaved Caspase‐3 antibody were counterstained with 0.2% toluidine blue in H_2_O, and sections with RANKL antibody with hematoxylin–eosin. Negative controls were performed without primary antibodies. Total number of Cleaved Caspase‐3‐positive osteocytes over bone area (N.Casp+/B.Ar n mm^−2^) and total number of Cleaved Caspase‐3‐positive osteocytes over total number of vital osteocytes (N.Casp+/N.Ot%), and total number of RANKL‐positive osteocytes over bone area (N.RANKL+/B.Ar n mm^−2^) were calculated.

### 2.9 Statistical analysis

Data are presented as mean ± standard error of the mean (SEM). Data analysis and statistical analysis were performed using GraphPad Prism 5 software (GraphPad Software, La Jolla, CA, USA) and IBM SPSS 23 statistical software (CircleCI, San Francisco, CA, USA). The number of cases in this study (8–10 cases per group) was based on our own studies and previously published studies^35^, ^36^, ^37^ that presented histological evaluation of bone biopsies. We could not carry out a power analysis, since we did not choose one specific parameter to compare the groups. Also, a direct comparison between the two graft types was not done before, so we decided to perform a multi‐parameter evaluation to identify potential differences in an unbiased manner. This study observed multiple parameters in the biopsies of the two bone graft types. Biopsies from all treated patients were compared between the retromolar and tuberosity bone groups. An unpaired two‐tailed Student's *t*‐test was performed to test differences in age and residual bone height between patients with retromolar and tuberosity bone grafts. No statistical differences were observed.

An unpaired nonparametric Mann–Whitney *U* and Pearson's chi‐squared test was performed to test differences between retromolar and tuberosity bone biopsies per region of interest. A paired Wilcoxon signed rank and McNemar test was performed to assess to test the different parameters between the different regions of interests within each bone group. Statistical significance was considered, if *p*‐values were <0.05.

## 3 RESULTS

In retromolar bone biopsies, compared to tuberosity bone biopsies, a higher bone volume (B.Ar/T.Ar%; mean ± SEM) in the center (RII: retromolar: 58% ± 8%; tuberosity: 16% ± 3%) and at the cranial side (RIII: retromolar: 60% ± 10%; tuberosity: 22% ± 5%) of the grafted area was observed (*p* < 0.05; Figure 2A–C). The other regions showed no significant differences in bone volume between biopsies with retromolar (native bone [NB]: 32% ± 5%; transition zone (TZ): 28% ± 9%; RI: 45% ± 8%) and tuberosity (NB: 24% ± 4%; TZ: 5% ± 2%; RI: 23% ± 9%) bone grafts (Figure 2C). There was a trend towards higher bone volume in regions towards the cranial side in retromolar bone grafts (*p =* 0.06; Figure 2C). There was no difference between the three regions (RI–RIII) in tuberosity bone grafts.

In retromolar bone biopsies, compared to tuberosity bone biopsies, less osteoid volume (O.Ar/B.Ar%; mean ± SEM) in the center (RII: retromolar: 3% ± 1%; tuberosity: 13% ± 3%) and at the cranial side (RIII: retromolar: 1% ± 1%; tuberosity: 11% ± 4%) was found (*p* < 0.05; Figure 2A,B,D). The other regions showed no differences in osteoid volume between retromolar (NB: 6% ± 3%; TZ: 11% ± 8%; RI: 10% ± 7%) and tuberosity (NB: 1% ± 1%; TZ: 4% ± 2%; RI: 5% ± 3%) bone grafts (Figure 2D). Osteoid volume tended to increase towards the cranial side of the biopsies within tuberosity bone grafts (*p* = 0.06), but was similar in the biopsies with retromolar grafts (Figure 2D).

**FIGURE 3:**
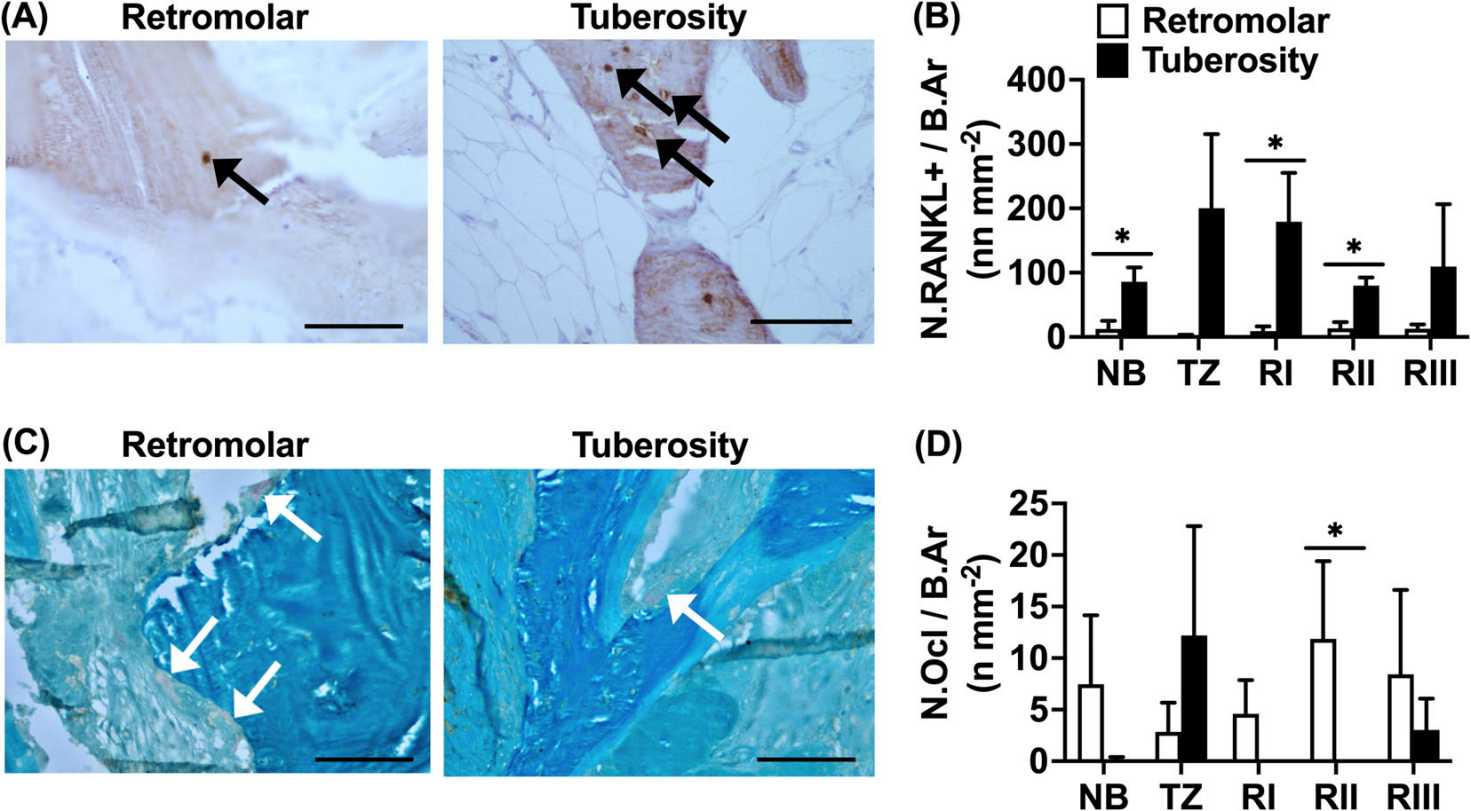
Total number of receptor activator of nuclear factor‐κB ligand (RANKL)‐positive osteocytes, and tartrate‐resistant acid phosphatase (TRAcP)‐positive osteoclasts in biopsies taken after maxillary sinus floor elevation (MSFE) with retromolar or tuberosity bone graft. (A) To visualize and calculate the number of RANKL‐positive osteocytes within the biopsies, consecutive sections were RANKL‐stained (brown) in biopsies taken after MSFE with retromolar or tuberosity bone graft. Images illustrate less RANKL‐positive osteocytes in retromolar than in tuberosity bone biopsies (RI region). Black arrows: RANKL‐positive cells. (B) Total number of RANKL‐positive osteocytes over bone area (N.RANK+/B.Ar n mm^−2^) was assessed for NB, TZ, RI, Rll, and Rlll. (C) To visualize and calculate the number of osteoclasts within the biopsies, consecutive sections were TRAcP‐stained (red) in biopsies taken after MSFE with retromolar or tuberosity bone graft. Images illustrate more TRAcP‐positive osteoclasts in retromolar than in tuberosity bone biopsies (RIII region). White arrows: TRAcP‐positive osteoclasts. (D) Number of TRAcP‐positive osteoclasts over bone area (N.Ocl/B.Ar n mm^−2^) was assessed for NB, TZ, Rl, Rll, and Rlll. Values are mean ± SEM (*n* = 3). *Significantly different between retromolar and tuberosity bone graft, *p <* 0.05. NB, native bone; RI, Region I; RII, Region II; RIII, Region III; TZ, transition zone. Magnification: 200x. Scale bar: 100 μm.

In retromolar bone biopsies, compared to tuberosity bone biopsies, a lower total number of RANKL‐positive osteocytes per bone area (N.RANKL+/B.Ar n mm^−2^; mean ± SEM) in residual native bone (NB: retromolar: 13 ± 13; tuberosity: 86 ± 23), at the caudal side (RI: retromolar: 9 ± 8; tuberosity: 179 ± 76) and in the center (RII: retromolar: 14 ± 9; tuberosity: 80 ± 12) of the grafted area (*p <* 0.05) was found, but values seemed different (not statistically significant) for the other regions (retromolar: TZ: 2 ± 2; RIII: 13 ± 7; tuberosity: TZ: 200 ± 115; RIII: 110 ± 97; Figure 3A,B). Moreover, no significant differences in total number of RANKL‐positive osteocytes per bone area were found between the regions per bone graft (Figure 3B).

In retromolar bone biopsies, compared to tuberosity bone biopsies, a higher total number of TRAcP‐positive osteoclasts per bone area (N.Ocl/B.Ar n mm^−2^; mean ± SEM) in the center (RII: retromolar: 12 ± 8; tuberosity: 0) of the grafted area was found (*p <* 0.05; Figure 3C,D), but the total number of TRAcP‐positive osteoclasts per bone area was similar for the other regions (retromolar: NB: 7 ± 7; TZ: 3 ± 3; RI: 5 ± 3; RIII: 8 ± 8; tuberosity: NB: 0; TZ: 12 ± 11; RI: 0; RIII: 3 ± 3; Figure 3D). Moreover, no significant differences in total number of TRAcP‐positive osteoclasts per bone area were found between the regions per bone graft (Figure 3D).

**FIGURE 4:**
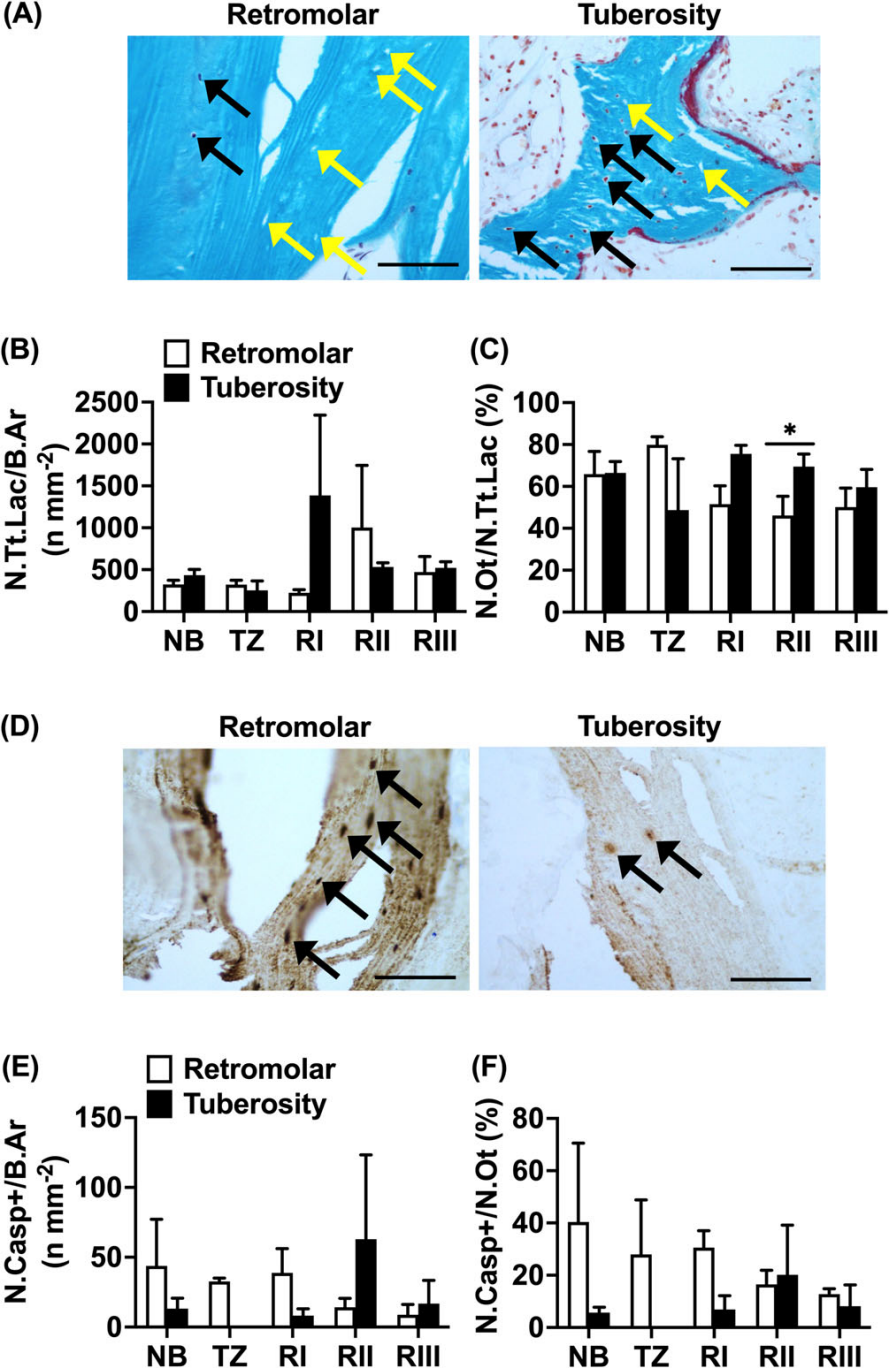
Total number of osteocytes, lacunae, and apoptotic osteocytes in biopsies taken after maxillary sinus floor elevation (MSFE) with retromolar or tuberosity bone graft. (A) Number of osteocytes (black arrows) and empty osteocyte lacunae (yellow arrows) were calculated in biopsies stained with Goldner's trichome taken after MSFE with retromolar and tuberosity bone graft. Images illustrate less osteocytes and more empty osteocyte lacunae in retromolar than in tuberosity bone biopsies (RII region). (B) Total number of lacunae over bone area (N.Tt.Lac /B.Ar n mm^−2^), and (C) Total number of osteocytes over total number of lacunae (N.Ot/N.Tt.Lac%) were assessed for NB, TZ, Rl, Rll, and Rlll. (D) To visualize and calculate apoptotic osteocytes within the biopsies, consecutive sections were stained with Cleaved Caspase‐3 (black) in biopsies taken after MSFE with retromolar or tuberosity bone graft. Black arrows: Cleaved Caspase‐3 positive apoptotic osteocytes. Images illustrate more Caspase‐3‐positive apoptotic osteocytes in retromolar than in tuberosity bone biopsies (RI region). (E) Total number Cleaved Caspase‐3‐positive osteocytes over bone area (N.Casp+/B.Ar n mm^−2^), and (F) Total number of Cleaved Caspase‐3‐positive osteocytes over total number of osteocytes (N.Casp+/N.Ot%) were assessed for NB, TZ, Rl, Rll, and Rlll. Values are mean ± SEM (*n* = 3–9). *Significantly different between retromolar and tuberosity bone graft, *p <* 0.05. B.Ar, bone area; NB, native bone; N.Casp+, number of Cleaved Caspase‐3‐positive osteocytes; N.Ot, number of osteocytes; N.Tt.Lac, total number of lacunae; RI, Region I; RII, Region II; RIII, Region III; TZ, transition zone. Magnification: 200x. Scale bar: 100 μm.

The total number of osteocyte lacunae per bone area (N.Tt.Lac/B.Ar n mm^−2^; mean ± SEM) was similar between biopsies with retromolar (NB: 324 ± 50; TZ: 323 ± 52; RI: 225 ± 43; RII: 1002 ± 954; RIII: 472 ± 215) and tuberosity bone grafts (NB: 433 ± 70; TZ: 254 ± 113; RI: 1386 ± 960; RII: 533 ± 51; RIII: 521 ± 72; Figure 4A,B). The total number of lacunae was also similar between the different regions per bone graft (Figure 4B).

In retromolar bone biopsies, compared to tuberosity bone biopsies, a lower total number of osteocytes per total number of osteocyte lacunae (N.Ot/N.Tt.Lac%; mean ± SEM) was observed in the center (RII: retromolar: 46 ± 9%; tuberosity: 69 ± 6%) of the grafted area (*p <* 0.05). The total number of osteocytes was similar in the other regions (retromolar: NB: 66% ± 11%; TZ: 80% ± 4%; RI: 52% ± 9%; RIII: 50% ± 9%; tuberosity: NB: 66% ± 5%; 49% ± 25%; TZ: 76% ± 4%; RI: 76% ± 4%; RIII: 60% ± 9%; Figure 4A,C). In retromolar bone biopsies, a lower total number of osteocytes in the grafted area (RI, RII, RIII) was observed than in NB (*p <* 0.05; Figure 4C). Also, a lower total number of osteocytes was seen in the center (RII) and cranial side (RIII) of the grafted area than in the transition zone (TZ; *p <* 0.05; Figure 4C). There were no significant differences in total number of osteocytes between the different regions in biopsies with tuberosity bone grafts.

Immunohistochemical staining of Cleaved Caspase‐3, a marker for cells that are undergoing apoptosis, showed a similar total number of apoptotic osteocytes per bone area (N.Casp+/B.Ar n mm^−2^; mean ± SEM) in both retromolar and tuberosity bone biopsies (retromolar: NB: 44 ± 33; TZ: 33 ± 2; RI: 39 ± 17; RII: 14 ± 6; RIII: 9 ± 7; tuberosity: NB: 13 ± 7; TZ: 0 ± 0; RI: 8 ± 5; RII: 63 ± 60; RIII: 17 ± 17; Figure 4D,E). Total number of apoptotic osteocytes was also similar in the different regions per bone graft (Figure 4D,E). In retromolar and tuberosity bone a similar percentage of osteocytes was apoptotic (N.Casp+/N.Ot%, mean ± SEM*)* (retromolar: NB: 40% ± 30%; TZ: 28% ± 21%; RI: 31% ± 6%; RII: 17% ± 5%; RIII: 13% ± 2%; tuberosity: NB: 6% ± 2%; TZ: 0% ± 0%; RI: 7% ± 5%; RII: 20% ± 19%; RIII: 8% ± 8%; Figure 4D,F). There were no significant differences in percentage apoptotic osteocytes in the different regions per bone graft (Figure 4F).

**FIGURE 5:**
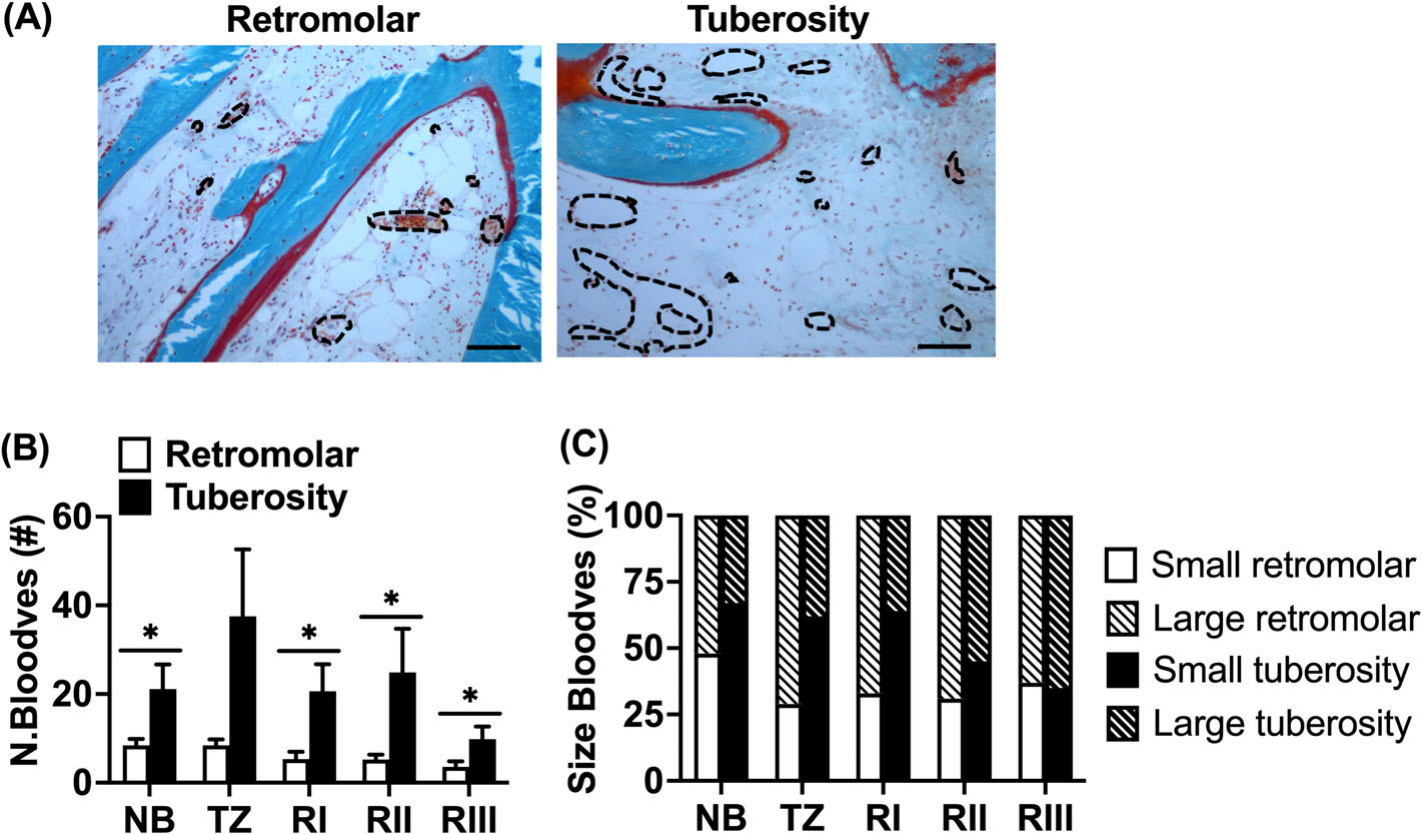
Total number of large‐ and small‐sized blood vessels in biopsies taken after maxillary sinus floor elevation (MSFE) with retromolar or tuberosity bone graft. (A) Blood vessel number was calculated in biopsies stained with Goldner's trichome taken after MSFE with retromolar or tuberosity bone graft. Images illustrate less blood vessels in retromolar than in tuberosity bone biopsies (RII region). Black dotted circumferential line: blood vessel. (B) Total number of blood vessels (N.Bloodves), and (C) Percentage of large‐ and small‐sized blood vessels was assessed for NB, TZ, Rl, Rll, and Rlll. Values are mean ± SEM (*n* = 3–9). *Significantly different between retromolar and tuberosity bone graft, *p <* 0.05. NB, native bone; N.Bloodves, number of blood vessels; RI, region I; RII, region II; RIII, region III; TZ, transition zone. Magnification: 100×. Scale bar: 100 μm.

In retromolar bone biopsies, compared to tuberosity bone biopsies, a lower total number of blood vessels (N.bloodves; mean ± SEM) in residual native bone (retromolar: NB: 8 ± 1; tuberosity: NB: 21 ± 6) and in the grafted area (retromolar: RI: 5 ± 2; RII: 5 ± 1; RIII: 4 ± 1; tuberosity: RI: 21 ± 6; RII: 25 ± 10; RIII: 10 ± 3) was observed (*p <* 0.05; Figure 5A,B), but was similar for the transition zone (retromolar: TZ: 8 ± 1; tuberosity: TZ: 38 ± 15; Figure 4B). In retromolar bone biopsies, the total number of blood vessels was positively correlated with osteoid volume in the grafted area (RI‐RIII; *r =* 0.43, *p* < *0*.05). In retromolar bone biopsies, compared to tuberosity bone biopsies, a higher percentage of large‐sized blood vessels in native bone (NB: retromolar: 52%; tuberosity: 33%), transition zone (TZ: retromolar: 71%; tuberosity: 38%), at the caudal side (RI: retromolar: 67%; tuberosity: 36%), and in the center (RII: retromolar: 69%; tuberosity: 55%) of the grafted area was observed (*p <* 0.05), but values were similar at the cranial side of the grafted area (RIII: retromolar: 63%; tuberosity: 65%; Figure 5C). In retromolar bone biopsies a higher percentage of large‐sized blood vessels, and a lower percentage of small‐sized blood vessels was shown in the transition zone and grafted area (large‐sized blood vessels: TZ: 71%; RI: 67%; RII: 69%; RIII: 63%) than in the residual native bone area (NB: 52%; *p <* 0.05; Figure 5C). Moreover, in RII (69%) compared to RIII (63%) there was a higher percentage of large‐sized blood vessels, and a lower percentage of small‐sized blood vessels (*p <* 0.05; Figure 5C). In tuberosity bone biopsies, a higher percentage of large‐sized blood vessels, and a lower percentage of small‐sized blood vessels was found in the grafted area (large‐sized blood vessels: RII: 55%; RIII: 65%) than in the residual native bone area (NB: 33%; *p <* 0.05; Figure 5C). Moreover, in tuberosity bone biopsies, the percentage of large‐sized blood vessels was increasing, and the percentage of small‐sized blood vessels was decreasing, in the grafted area from the caudal towards the cranial side of the biopsy (large‐sized blood vessels: RI: 36%; RII: 55%; RIII: 65%; small‐sized blood vessels: RI: 64%; RII: 45%; RIII: 35%; *p <* 0.05; Figure 5C).

## 4 DISCUSSION

Four months after MFSE, differences in bone vitality and vascularization were observed between retromolar and tuberosity bone grafts. Compared to tuberosity bone biopsies, retromolar bone biopsies showed: (i) 40% higher bone volume (B.Ar/T.Ar%) in the grafted RII and RIII regions; (ii) 10% lower osteoid volume (O.Ar/B.Ar%) in these two regions; (iii) 90% lower a total number of RANKL‐positive osteocytes in the grafted area (caudal side [RI] and center [RII]); (iv) higher a total number of osteoclasts in region RII of the grafted area; (v) 23% lower total number of osteocytes per total number of osteocyte lacunae (N.Ot/N.Tt.Lac%) in region RII; (vi) 80%–25% lower total number of blood vessels in the grafted area from RI to RIII; (vii) 31% (RI) and 14% (RII) higher number of large‐sized blood vessels in the grafted area; and (viii) similar total number of osteocytes, and total number of apoptotic osteocytes.

Since our results showed more osteoid formation and a higher number of blood vessels in the grafted areas of patients who underwent MSFE with tuberosity bone grafts compared to patients with retromolar bone grafts, we postulate that tuberosity bone grafts might result in faster bone regeneration than retromolar bone grafts in MSFE.

Moreover, retromolar bone biopsies showed higher bone volume in the grafted area than tuberosity bone biopsies. These findings may be explained by a higher mineralization degree of the original graft, as retromolar bone grafts are predominantly composed of cortical bone, and tuberosity bone grafts of cancellous bone.^21^, ^38^ A slower bone remodeling process of cortical bone versus cancellous bone is expected.^21^, ^38^ Therefore, our observation that significant differences in the mineralized bone area are present in our biopsies, likely results from the original composition of the bone graft and not yet from new bone formation. Since the bone biopsies in the present study were retrieved 4 months after MSFE, differences in the mineralized bone area due to bone remodeling between the two graft materials may be leveled in the long term, if graft remodeling has occurred to a high degree. Apoptotic osteocytes and empty lacunae were observed in both types of bone grafts, and therefore bone remodeling will likely continue after the biopsy retrieval (4‐month time point). This is in line with findings by others showing that the bone mineralization degree in cortical bone grafts (chin and retromolar area), but not in corticocancellous bone grafts, decreases during 6 months post‐MSFE period.^25^, ^38^ However, 6 months post‐MSFE the bone mineralization degree is still higher in cortical than corticocancellous bone grafts, indicating that both graft origin and remodeling rate influence the mineralization degree.^25^, ^38^

In this study, lower osteoid volume at the cranial and center of the retromolar bone‐grafted areas, compared to tuberosity bone‐grafted areas, was found. The reason for this observation is currently unexplained. Interestingly, osteoid volume increased towards the cranial side of the grafted area, which may have resulted from active bone formation starting from the cranial side of the biopsy. This is in line with earlier observations that bone formation may start not only from the maxillary native bone, but from the cranial side as well.^29^, ^35^ Moreover, it has been shown that the Schneiderian membrane of the maxillary sinus, which is lifted during MSFE to insert the graft material, contains a cell population with potential for osteogenic differentiation.^40^

We found similar total numbers of lacunae and apoptotic osteocytes in biopsies from retromolar and tuberosity bone‐grafted areas. Remarkably, we observed a 90% lower total number of RANKL‐positive osteocytes at the caudal side and in the center of the grafted area, and a higher number of TRAcP‐positive osteoclasts in the center of the grafted area of retromolar vs. tuberosity bone biopsies. Osteocytes embedded in bone have been postulated to orchestrate bone homeostasis by regulating both bone‐forming osteoblasts and bone‐resorbing osteoclasts.^41^, ^42^ RANKL expression by osteocytes is an important signal to recruit osteoclasts.^43^, ^44^ RANKL, a transmembrane protein from the tumor necrosis factor (TNF) superfamily, is known to play a central role in osteoclastogenesis.^45^ Therefore, the lower number of RANKL‐positive osteocytes observed in retromolar bone biopsies in our study, may indicate less active bone remodeling in retromolar than in tuberosity bone grafts.

Bone is highly vascularized, and vascular development needs to be induced prior to osteogenesis. Our results showed that the total number of blood vessels in the grafted area was lower in retromolar versus tuberosity bone biopsies, which was accompanied by a lower percentage of osteoid volume. In contrast, the higher percentage of small‐sized blood vessels and the lower percentage of large‐sized blood vessels as we observed in the tuberosity bone‐grafted areas indicates higher angiogenic activity in tuberosity bone graft. This is in agreement with earlier studies showing that bone formation is related to increased blood vessel formation.^29^, ^39^ The lower total number of osteocytes in the center of the grafted area of the retromolar grafts may consequently be the result of reduced diffusion of oxygen and nutrients due to delayed vascularization in these grafts. This confirms findings in earlier studies, that is, that cortical bone grafts, compared to cancellous bone grafts, show delayed vascularization due to lack of porosity and consequent inhibition of vascular ingrowth.^21^

The study was conducted retrospectively resulting in several limitations. A limitation of the present study was that we compared two different autologous bone grafts in patients undergoing unilateral MSFE. To exclude inter‐patient variation, a bilateral sinus floor elevation model would be more appropriate to compare two different grafting materials. Another limitation of this study was that we only analyzed biopsies at one time point, preventing to assess the dynamics of the remodeling process in both types of bone grafts. Therefore, we can only deduce that retromolar grafts displayed a slower bone remodeling rate, but cannot rule out that remodeling might reach similar levels at a later time point. Another limitation of this study was the use of two bone harvesting techniques. However, the bone harvesting techniques were unlikely to affect the results of our study in terms of bone vitality of the graft, since the harvesting techniques were as “atraumatic” as possible. This appears to be histologically confirmed since we did not observe necrotic bone tissue. A limitation of this study was also that no follow‐up data of the patients could be obtained. However, no complaints regarding all dental implants have been reported thus far.

In summary, we found that the use of tuberosity bone graft in human MSFE resulted in a 10% higher osteoid volume in the center and at the cranial side of the grafted area, and 150%–300% higher total number of blood vessels in the total grafted area compared to retromolar bone grafts. We conclude that tuberosity bone grafts showed enhanced bone vitality and vascularization in patients undergoing MSFE in comparison with retromolar bone grafts, either due to a faster bone remodeling rate or due to an earlier start of bone remodeling in tuberosity bone graft‐treated patients. Based on our histological data, it appears that tuberosity bone might perform better as an autologous graft material in MSFE than retromolar bone, since more osteoid was deposited, more blood vessels were formed, and a more active remodeling process was initiated. A shorter healing period before dental implant placement and loading might be feasible, if tuberosity bone grafts are used.

## AUTHOR CONTRIBUTIONS

**Vivian Wu**: Conceptualization and design (lead), acquisition of data (lead), analysis and interpretation of data (lead), drafting the manuscript (lead), given final approval of the version to be published, agreed to be accountable for all aspects of the work. **Engelbert A. J. M. Schulten**: Conceptualization and design (equal), acquisition of data (equal), analysis and interpretation of data (equal), revising the manuscript (equal), given final approval of the version to be published, agreed to be accountable for all aspects of the work. **Marco N. Helder**: Conceptualization and design (equal), acquisition of data (equal), analysis and interpretation of data (equal), revising the manuscript (equal), given final approval of the version to be published, agreed to be accountable for all aspects of the work. **Christiaan M. ten Bruggenkate**: Conceptualization and design (equal), acquisition of data (equal), analysis and interpretation of data (equal), revising the manuscript (equal), given final approval of the version to be published, agreed to be accountable for all aspects of the work. **Nathalie Bravenboer**: Conceptualization and design (equal), acquisition of data (equal), analysis and interpretation of data (equal), revising the manuscript (equal), given final approval of the version to be published, agreed to be accountable for all aspects of the work. **Jenneke Klein‐Nulend**: Conceptualization and design (equal), acquisition of data (equal), analysis and interpretation of data (equal), revising the manuscript (equal), given final approval of the version to be published, agreed to be accountable for all aspects of the work.

## CONFLICT OF INTEREST

The authors declare no conflict of interest.

## Data Availability

The data that support the findings of this study are available on request from the corresponding author. The data are not publicly available due to privacy or ethical restrictions.
